# Regulation of axonal regeneration by the level of function of the endogenous Nogo receptor antagonist LOTUS

**DOI:** 10.1038/s41598-017-12449-6

**Published:** 2017-09-21

**Authors:** Tomoko Hirokawa, Yixiao Zou, Yuji Kurihara, Zhaoxin Jiang, Yusuke Sakakibara, Hiromu Ito, Kengo Funakoshi, Nobutaka Kawahara, Yoshio Goshima, Stephen M. Strittmatter, Kohtaro Takei

**Affiliations:** 1 Molecular Medical Bioscience Laboratory, Yokohama City University Graduate School of Medical Life Science, Yokohama, 230-0045 Japan; 2 Cellular Neuroscience, Neurodegeneration and Repair Program, Departments of Neurology and Neuroscience, Yale University School of Medicine, New Haven, CT 06536 USA; 3 Department of Molecular Pharmacology and Neurobiology, Yokohama City University Graduate School of Medicine, Yokohama, 246-0004 Japan; 4 Department of Neuroanatomy, Yokohama City University Graduate School of Medicine, Yokohama, 246-0004 Japan; 5 Department of Neurosurgery, Yokohama City University Graduate School of Medicine, Yokohama, 246-0004 Japan; 6 Advanced Medical Research Center, Yokohama City University Graduate School of Medicine, Yokohama, 236-0004 Japan

## Abstract

Axonal regeneration in the adult mammalian central nervous system is limited in part by the non-permissive environment, including axonal growth inhibitors such as the Nogo-A protein. How the functions of these inhibitors can be blocked remains unclear. Here, we examined the role of LOTUS, an endogenous Nogo receptor antagonist, in promoting functional recovery and neural repair after spinal cord injury (SCI), as well as axonal regeneration after optic nerve crush. Wild-type untreated mice show incomplete but substantial intrinsic motor recovery after SCI. The genetic deletion of LOTUS delays and decreases the extent of motor recovery, suggesting that LOTUS is required for spontaneous neural repair. The neuronal overexpression of LOTUS in transgenic mice promotes motor recovery after SCI, and recombinant viral overexpression of LOTUS enhances retinal ganglion cell axonal regeneration after optic nerve crush. Thus, the level of LOTUS function titrates axonal regeneration.

## Introduction

Neurons in the central nervous system (CNS) undergo limited axonal regeneration after trauma, in part because of the non-permissive environment^1,2^. The CNS environment includes axonal growth inhibitors (AGIs) derived from myelin, such as Nogo protein^3^, myelin-associated glycoprotein (MAG)^4^, and oligodendrocyte myelin glycoprotein (OMgp)^5^. AGIs are also derived from glial components, such as chondroitin sulfate proteoglycan^6,7^ and B lymphocyte stimulator (BLyS), which is a tumour necrosis factor superfamily member expressed in CNS astrocytes^8^. Each of these five AGIs binds to Nogo receptor-1 (NgR1) and induces nerve growth cone collapse and neurite outgrowth inhibition^1,2,5,8–11^. NgR1 forms a receptor complex with leucine-rich repeat and immunoglobulin domain-containing Nogo receptor-interacting protein-1^12^ and either the 75-kDa neurotrophin receptor^13^ or tumour necrosis factor receptor superfamily member 19^14^.

These co-receptors play a role in intracellular transduction and mediate actin depolymerization through activation of RhoA and Rho-associated, coiled-coil containing protein kinase^11–15^. Therefore, NgR1 is considered to be a promising therapeutic target for axonal regeneration. In fact, accumulating evidence has shown that an NgR1 antagonist peptide that is specifically competitive with Nogo^16^ or genetic deletion of NgR1^17^ enhances axonal regeneration after spinal cord injury (SCI). Similarly, a soluble fragment of NgR1 containing a ligand-binding site can be used as a decoy-like protein to inhibit all five AGIs^18–21^ and is effective in promoting function even in chronic contusion injury^22^. Furthermore, triple genetic deletion of Nogo, MAG, and OMgp produces greater improvement in axonal regrowth following SCI compared with a single Nogo mutation^23^. These reports suggest that inhibition of the function of the multiple glial components that bind to NgR1 may improve the ability of neurons to regenerate their damaged CNS axons more effectively. Whether inhibition of BLyS function contributes to axonal regeneration *in vivo* remains unknown.

Recently, an endogenous NgR1 antagonist, lateral olfactory tract usher substance (LOTUS)/cartilage acidic protein-1B (Crtac1B) was identified in the developing brain and shown to contribute to axon tract formation by antagonizing Nogo-induced NgR1 function^24^. The carboxyl-terminal region of LOTUS binds to NgR1^25^ and blocks the binding of four AGIs (Nogo, MAG, OMgp, BLyS) to NgR1 as well as their axonal growth inhibition^26^. Therefore, LOTUS is a potent endogenous inhibitor of NgR1 function. Here, we examined whether LOTUS contributes to functional recovery following SCI using *lotus*-deficient and *lotus-*overexpressing transgenic mice. We also provide evidence showing that LOTUS plays a crucial role in neuronal regeneration following SCI.

## Results

### LOTUS is required for recovery of neurological function after SCI

**Figure 1:**
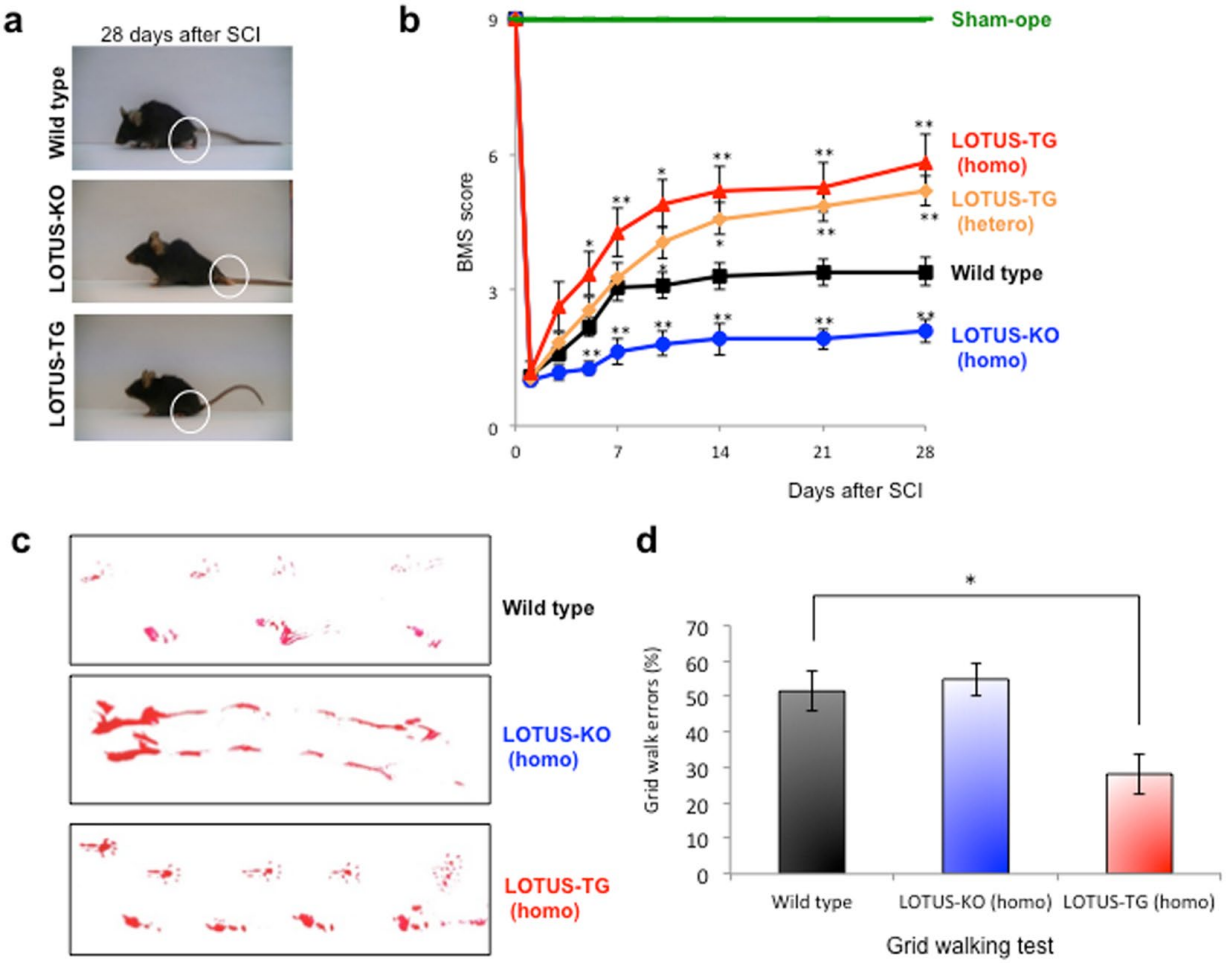
Locomotor activity after SCI (dorsal hemitransection) in mice lacking or overexpressing LOTUS. (**a**) Photographs of WT (Lotus+/+), LOTUS-KO (Lotus−/−), and LOTUS-TG mice at 4 weeks after SCI. The white circles indicate the typical hind limb phenotype. (**b**) Functional analysis of open field locomotor activity as shown with BMS scoring up to 28 days after SCI in sham-operated (n = 4), WT (Lotus+/+, n = 27), LOTUS-TG (heterozygous, n = 20), LOTUS-TG (homozygous, n = 11), and LOTUS-KO (homozygous, n = 11) mice. Note that the locomotion delay in LOTUS-KO mice and the locomotion recovery in LOTUS-TG (homozygous) mice were significant beginning at early stages after SCI. The quantitative data are shown as the mean ± SEM. **p < 0.01 and *p < 0.05 compared with scores in WT mice. The statistical analysis was performed with the Mann-Whitney U-test. (**c**) Footprints in WT, LOTUS-KO (homozygous), and LOTUS-TG (homozygous) mice. (**d**) Grid walking test in WT (n = 13), LOTUS-KO (homozygous, n = 4), and LOTUS-TG (homozygous, n = 10) mice. Note that the locomotion recovery in LOTUS-TG was significant. The quantitative data are shown as the mean ± SEM. *p < 0.05 compared with scores in WT mice. The statistical analysis was performed with one-way ANOVA with the Tukey test.

Mice and rats show incomplete but substantial spontaneous motor recovery after SCI. We first examined whether LOTUS is involved in functional motor recovery after SCI using *lotus*-deficient (knock out; LOTUS-KO) mice. We generated LOTUS-KO mice, which show no difference in body weight (Fig. S1a,b) and normal open field locomotor activity relative to controls (Fig. S1c). However, abnormal development of the lateral olfactory tract was observed in LOTUS-KO mice^24^. To investigate neural repair after CNS trauma, we employed a dorsal over-hemitransection model in which the spinal cord of wild-type (WT) or LOTUS-KO mice was cut 1 mm deep from the dorsal surface at the thoracic (T)8 level. We confirmed no difference in the lesion level 1 day after SCI between WT and LOTUS-KO mice (Fig. S2c,e). We then analysed locomotor activities as scored by the Basso Mouse Scale (BMS)^27^, footprints during locomotion, and the grid-walking test. The BMS open field test was employed to evaluate hind limb motor function, and the locomotor activities were analysed using only mice showing scores of 1 at 1 day after SCI. As shown in Fig. 1, motor improvement was observed through 7 days after SCI in WT mice, but the graph of the BMS locomotion score in WT mice showed no further increase of motor recovery thereafter. At 28 days after SCI, the WT mice reached a score of 3 or 4 and showed occasional, frequent, or consistent dorsal stepping; however, plantar stepping was either occasional or absent (Fig. 1a). By contrast, LOTUS-KO mice obtained a lower performance level, as indicated by a score of 1 or 2, and showed slight or extensive ankle movement (Fig. 1a). Furthermore, a delay in the rate of motor recovery was observed in the BMS locomotion score (Fig. 1b), and impaired locomotion was also observed with respect to footprints (Fig. 1c). Because neither WT nor LOTUS-KO mice showed plantar stepping, there was no significant difference in the grid-walking test (Fig. 1d).

**Figure 2:**
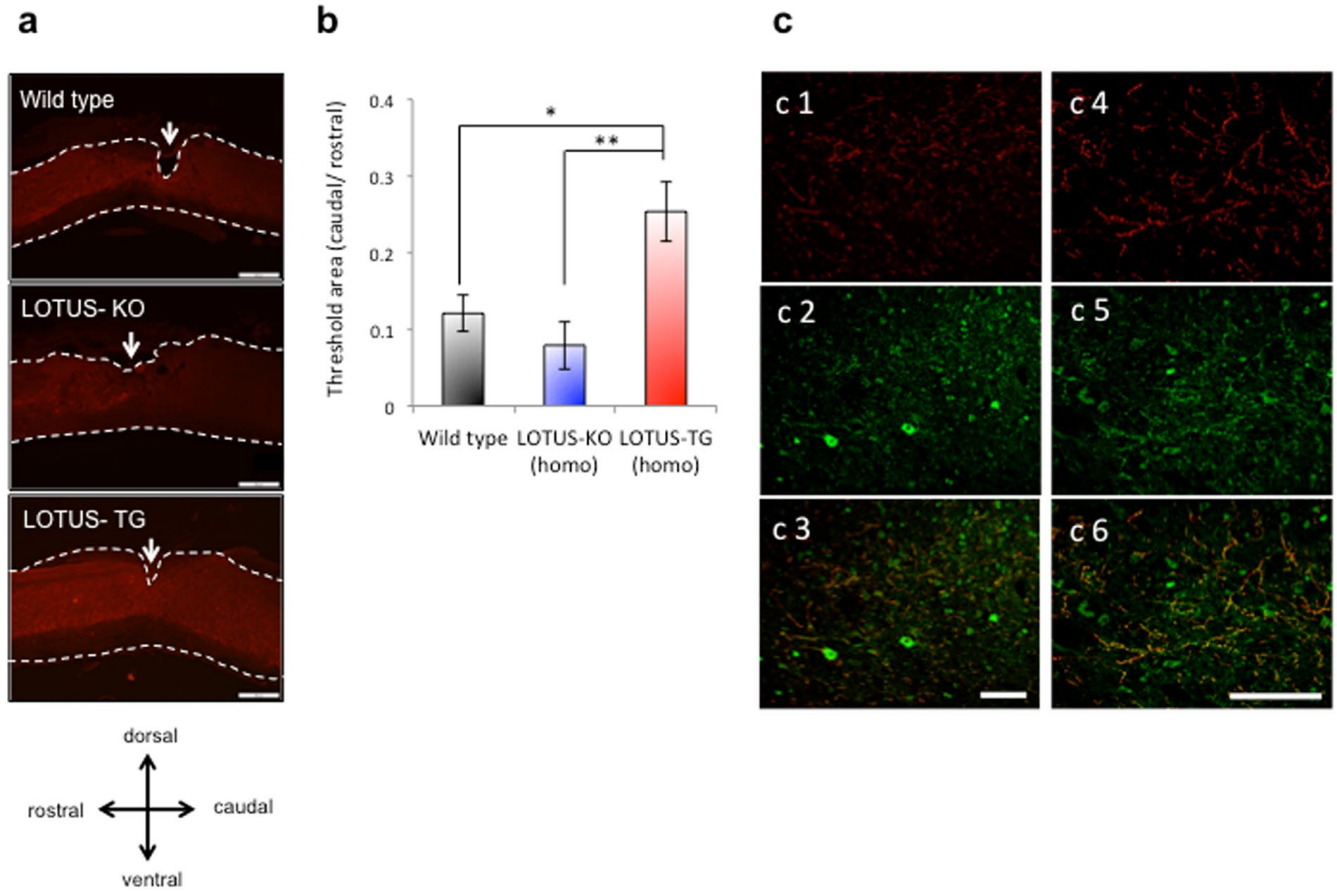
Axonal growth in the spinal cord in WT, LOTUS-KO, and LOTUS-TG mice after SCI. (**a**) Images from sagittal sections of 5-HT immunohistochemical staining revealed 5-HT-positive raphespinal tract axons caudal to the injury site in each group 4 weeks after SCI. The arrows indicate the injury site. The dashed lines indicate the border of the spinal cord. Scale bar: 500 μm. (**b**) Quantitative analysis of 5-HT immunoreactivity within 0.5 mm caudal to the injury site compared to that rostral to the injury site is shown as a relative ratio (caudal/rostral in each animal) of 5-HT immunoreactivity. *p < 0.05 compared with the ratio in WT mice (WT: n = 4; LOTUS-KO: n = 5; LOTUS-TG: n = 6). The quantitative data are shown as the mean ± SEM. The statistical analysis was performed with one-way ANOVA and the Tukey test. (**c**) Images from sagittal sections of double immunostaining for 5-HT (c1 and c4), HA-LOTUS (c2 and c5), and merged images (c3 and c6) revealed 5-HT-positive regenerating axons with overexpressed LOTUS (HA-LOTUS) caudal to the injury site in LOTUS-TG mice 4 weeks after SCI. The images in c4, c5, and c6 are shown at high magnification. Note the double-immunopositive fibres in the spinal cord caudal to the injury site after SCI. Scale bars: 100 µm.

**Figure 3:**
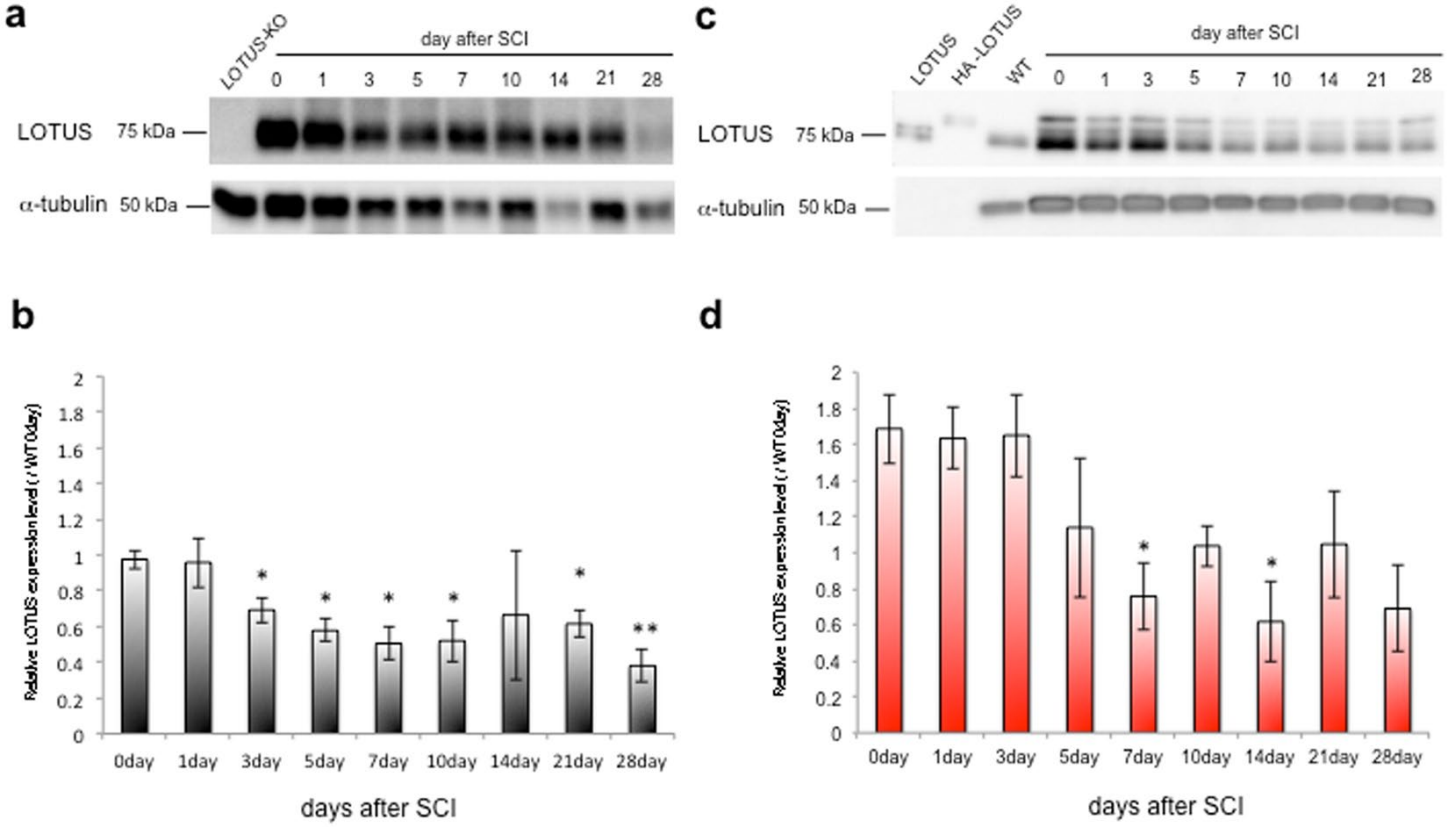
Time course of the expression of endogenous LOTUS and overexpressed HA-LOTUS in the injured region of the spinal cord after SCI. Immunoblots of endogenous LOTUS (**a**) and overexpressed HA-LOTUS (**c**) in the injured region of the spinal cord at 0, 1, 3, 5, 7, 10, 14, 21, and 28 days after SCI. α-Tubulin was used as an internal control protein. LOTUS and HA-LOTUS indicate input controls of recombinant LOTUS protein and HA-LOTUS protein, respectively. WT indicates brain protein lysate from an adult WT mouse. The expression levels of endogenous LOTUS (**b**) and overexpressed HA-LOTUS (**d**) were quantified by the intensity of protein immunoblotting and normalized to the observed intensity on day 0 relative to the SCI. The quantitative data are shown as the mean ± SEM. Significance was obtained by performing Student’s t-test (n = 4 experiments). **p < 0.01 and *p < 0.05 compared with the ratio at day 0 after SCI.

We further examined whether genetic loss of LOTUS led to decreased serotonergic projections caudal to the injury site. Descending serotonergic projections of the raphespinal tract in the ventral horn of the lumbar spinal cord were verified by immunohistochemistry with a 5-hydroxytryptamine (5-HT; serotonin) antibody 28 days after SCI. We then quantitatively analysed 5-HT immunoreactivity within 0.5 mm caudal to the injury site compared to expression rostral to the injury site. There was no difference in 5-HT immunoreactivity before the SCI and at 1 day after SCI between WT and LOTUS-KO mice (Fig. S2a–d). Immunohistochemistry using the 5-HT antibody revealed dense descending serotonergic projections in the ventral horn of WT mice, as shown by 5-HT-immunopositive axon fibres. By contrast, low 5-HT immunoreactivity was observed in the ventral horn of LOTUS-KO mice, with a tendency towards decreased serotonergic axon fibre density caudal to the injury site (Fig. 2a,b). Taken together, these data suggest that LOTUS is crucial for neural repair and recovery of function. Interestingly, the expression level of LOTUS in the injured region of the spinal cord was dramatically decreased beginning 3 days after SCI and remained at a lower level (an approximately 50% decrease) 7 days after SCI and thereafter (Fig. 3a,b). This low level of LOTUS expression corresponds well to the plateau of motor recovery measured by BMS for WT mice (Fig. 1b). This correlation between the LOTUS expression level and locomotor activity suggests that a decrease in LOTUS expression may suppress spontaneous motor recovery and that LOTUS expression is required for neurological recovery.

### LOTUS overexpression promotes retinal ganglion cell axon regrowth after optic nerve crush

**Figure 4:**
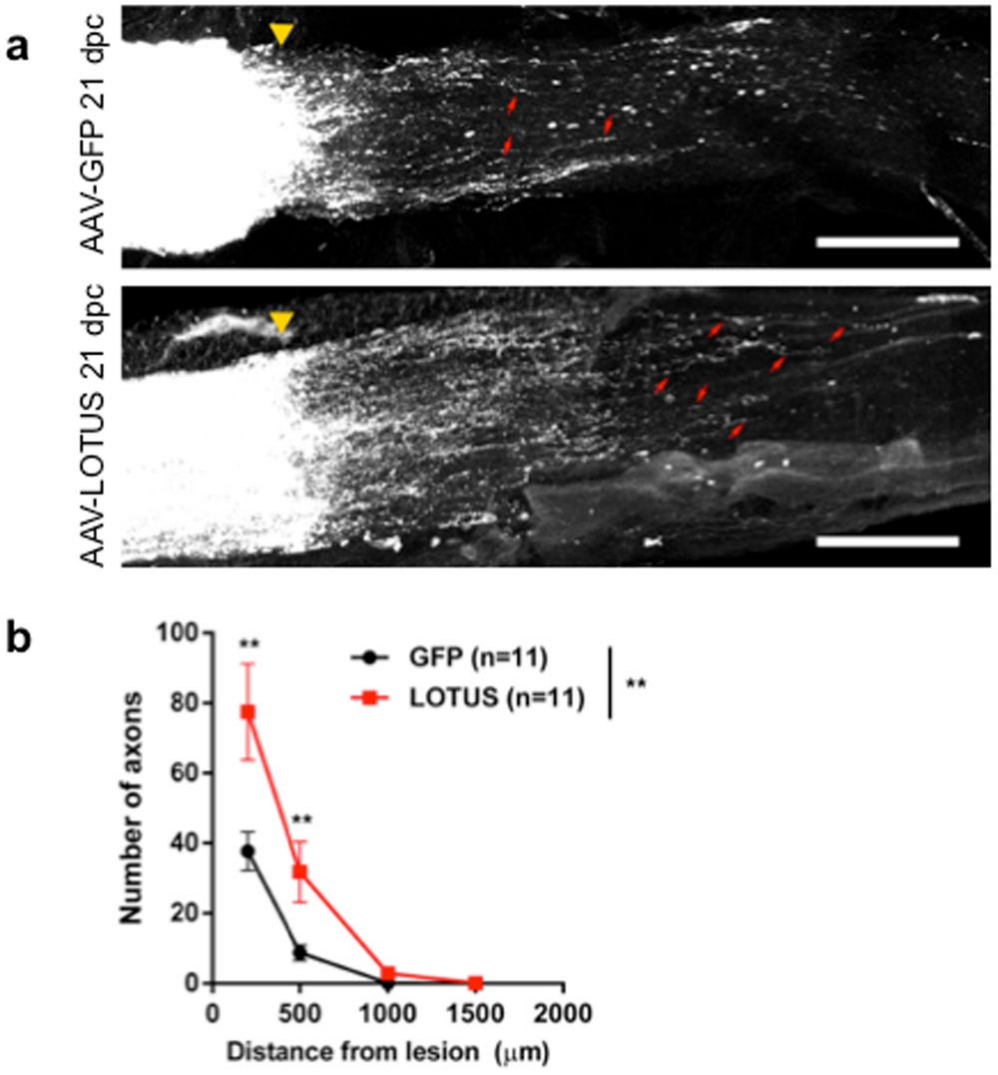
Enhanced RGC axon regeneration with LOTUS expression. (**a**) Representative images of optic nerves showing CTB-labelled RGC axons 21 days after injury in mice injected with AAV-GFP (top) or AAV-LOTUS (bottom). Scale bar: 200 µm. The yellow triangles indicate crush sites. The red arrows indicate regenerating RGC axons distal to crush sites. (**b**) Quantification of regenerating fibres at different distances distal to the lesion sites. Whole optic nerves were cleared, and confocal stacks were captured with 4-μm optic sections. All of the optic sections were quantified per animal. The quantitative data are shown as the mean ± SEM. A significant difference was found between the control and LOTUS-expressing groups (two-way ANOVA, **p < 0.01) and at 200 µm and 500 µm from the lesion (Fisher’s LSD post hoc test, **p < 0.01).

Because the loss of LOTUS reduced neural repair, we considered whether increased LOTUS might promote recovery. As a first step, we examined a simplified axonal regeneration model, that of retinal ganglion cell (RGC) axon growth after optic nerve crush injury. LOTUS overexpression in RGCs was obtained by intravitreal injection of a recombinant virus. To investigate regeneration, we crushed the optic nerve in WT mice 2 weeks after injection of an adeno-associated virus (AAV) containing green fluorescence protein (GFP) (AAV-GFP) as a control or an AAV containing LOTUS (AAV-LOTUS). We then analysed the optic nerve using anterograde tracing with fluorescence-conjugated cholera toxin subunit B (CTB) 21 days after the injury. Regenerating axons in the optic nerve were detected by counting CTB-positive axons that intersected vertical lines drawn at fixed distances (200, 500, 1000, and 1500 µm) distal to the crush site in longitudinal sections through the optic nerve. At distances of 200 and 500 µm, the number of CTB-positive axons were significantly increased in AAV-LOTUS-injected optic nerves compared with AAV-GFP-injected optic nerves (Fig. 4). The overexpression of LOTUS did not affect the survival of RGCs in optic nerve crush mouse model (Fig. S3). Thus, increased LOTUS expression enhanced CNS axon regeneration *in vivo*.

### Overexpression of LOTUS promotes functional recovery after SCI

As increased LOTUS expression promoted axon regeneration, we evaluated its effect after SCI. By driving LOTUS expression from the synapsin-1 promoter, we generated transgenic mice overexpressing LOTUS specifically in neurons (LOTUS-TG). We confirmed the overexpression of haemagglutinin (HA)-tagged LOTUS (HA-LOTUS) with immunoblotting (Fig. S4). The LOTUS-TG mice showed normal open field locomotor activity and no difference in body weight, similar to LOTUS-KO mice (Fig. S1). In contrast to LOTUS-KO mice, LOTUS overexpression in LOTUS-TG mice drastically enhanced motor recovery after SCI compared with WT mice, as determined by the BMS locomotion score (Fig. 1b), footprints (Fig. 1c) and the grid-walking test (Fig. 1d). Thus, intrinsic motor recovery was increased in LOTUS-TG mice (Fig. 1). At 28 days after SCI, the LOTUS-TG mice reached a higher performance level, as indicated by a score of 5 or 6, and showed occasional plantar stepping, or frequent or consistent plantar stepping with some coordination of paws parallel to the initial contact and lift off (Fig. 1a). Histological analysis revealed that 5-HT-immunopositive descending serotonergic axon fibres were increased in the spinal cord caudal to the injury site compared with WT mice 28 days after SCI (Fig. 2), although there was no difference between WT and LOTUS-TG mice in 5-HT immunoreactivity before or 1 day after SCI (Fig. S2). Moreover, overexpressed LOTUS (HA-LOTUS) co-localized with 5-HT-positive descending axon fibres in the caudal region of the spinal cord, as shown by HA-tag immunostaining (Fig. 2c). We further examined the expression of growth associated protein-43 (GAP-43), a nerve growth marker, in the spinal cord at 28 days after SCI. There was no difference in the intact spinal cord of LOTUS-TG mice compared to WT mice (data not shown), whereas the expression level of GAP-43 in LOTUS-TG mice was higher than in WT and LOTUS-KO mice (Fig. S5). Although the expression level of overexpressed HA-LOTUS in LOTUS-TG mice was decreased 5–7 days after SCI and remained at a lower level in the injured region (Fig. 3), the combined LOTUS expression level from the endogenous allele and the transgene was greater at all time points after SCI compared with WT mice. The onset of the decrease in LOTUS expression corresponded well to that of the plateau of motor recovery in WT mice (Fig. 1b). These behavioural and histological results support a model in which LOTUS overexpression promotes functional motor recovery after SCI.

## Discussion

The major conclusion of this study is that LOTUS levels are directly correlated with neural repair after SCI and optic nerve crush. In loss-of-function experiments, LOTUS-KO mice exhibit a decrease in motor recovery after generation of a dorsal over-hemitransection model of SCI. The level of motor recovery in heterozygous LOTUS-KO mice is intermediate between that of WT and homozygous LOTUS-KO mice (data not shown), suggesting that the level of motor recovery is dependent on the level of LOTUS expression. Interestingly, a 50% decrease in LOTUS expression from 7 days after SCI through later time points may lead to the decrease in the rate of motor recovery of WT mice at this stage (Fig. 3a,b). This observation is consistent with the hypothesis that motor recovery after SCI occurs in a manner that is dependent on the LOTUS expression level. Indeed, LOTUS-TG mice that overexpress LOTUS in neurons show a remarkably enhanced intrinsic motor recovery, as shown by a BMS locomotion score of over 4 points after dorsal over-hemitransection. This enhanced recovery occurs in an expression level-dependent manner, as evidenced by the finding that motor recovery in heterozygous LOTUS-TG mice was intermediate between of that WT and homozygous LOTUS-TG mice (Fig. 1b). Furthermore, the expression level of GAP-43 was higher than that in LOTUS-TG mice after SCI, and LOTUS overexpression did not affect the cell survival after optic nerve crush. These *in vivo* findings provide evidence that LOTUS is a neural repair-promoting factor. Notably, the injection of NEP-40 protein, a Nogo-66 antagonistic peptide, inhibits neural apoptosis and shows a neuroprotective effect in a rat ischaemic brain model, indicating that LOTUS may have neuroprotective abilities^28^. This hypothesis is consistent with our result that when compared with WT mice, LOTUS-TG mice show early functional improvements and signs of neuroprotection 5 days after SCI (Fig. 1b). In the visual system, LOTUS overexpression yields axonal regeneration after optic nerve crush in a cell autonomous manner. Thus, a therapeutic approach using LOTUS gene transfection may be useful for neuronal regeneration.

As LOTUS is a potent endogenous inhibitor of AGIs^26^, neurons expressing LOTUS may be able to overcome the inhibitory effect of AGIs on intrinsic axonal regeneration. However, the level of LOTUS expression in the injured spinal cord was decreased to 50% 7 days after SCI and at subsequent time points (Fig. 3b). The down-regulation of LOTUS expression is strongly correlated with the perturbation of intrinsic motor recovery after SCI (Fig. 1). Thus, the non-permissive environment for neuronal regeneration in the adult CNS may be due to both the presence of AGIs and the down-regulation of LOTUS expression in neurons. Indeed, regenerating axons after SCI in LOTUS-TG mice overexpressed LOTUS; thus, the maintenance of LOTUS expression may be important for overcoming the influence of AGIs. However, the LOTUS concentration in cerebrospinal fluid (CSF) is markedly decreased in patients with cerebral infarction (unpublished data) and multiple sclerosis^29^ compared with normal healthy controls. This phenomenon suggests that LOTUS expression in the CNS or secretion into the CSF is down-regulated by CNS damage and/or inflammation, although how LOTUS expression or secretion is down-regulated remains unknown. Thus, inhibiting the down-regulation of LOTUS may be a possible therapeutic approach for neuronal regeneration after SCI, as may the delivery of recombinant LOTUS protein to the injured region of the spinal cord.

The genetic deletion of NgR1 or soluble NgR1 enhances axonal regeneration after SCI by inhibiting all five NgR1 ligands (AGIs)^17–22^. Moreover, triple genetic deletion of three NgR1 ligands, Nogo, MAG, and OMgp, provides greater improvement in axonal regrowth following SCI compared with the single deletion of Nogo^23^. As LOTUS suppresses axon growth inhibition by blocking the interaction between NgR1 and four of its ligands^26^, LOTUS may be as effective as NgR1 deletion for overcoming NgR1-mediated limitations in the regeneration of neuronal function. Of note, LOTUS is an endogenous protein that is present in the healthy nervous system, and the expression level of LOTUS correlates with functional recovery after SCI. Therefore, supplying LOTUS protein may avoid any potential side effect of overcoming the influences of AGIs themselves and could be desirable as a future therapeutic approach for improving neural function.

In conclusion, the present study demonstrates that the deletion of a single protein, LOTUS, results in a remarkable delay in functional recovery after SCI, suggesting that this molecule promotes neuronal regeneration after SCI. The overexpression of LOTUS significantly promotes axon regrowth after optic nerve and spinal cord injuries and may therefore have potential for the clinical treatment of humans with CNS axonal injury.

## Methods

### Animals

#### Spinal cord injury model

C57BL/6J mice were purchased from Charles River Co. (Japan, Inc.), and the *lotus/crtac1b* mutant mice (Acc. No. CDB0599K, http://www.cdb.riken.jp/arg/mutant%20mice%20list.html) were generated as previously described^24^ (see http://www.cdb.riken.jp/arg/Methods.html). The *ngr1* mutant mice were generated as previously described^17^. LOTUS-overexpressing mice were generated using of the synapsin-1 promoter, which directs neuron-specific LOTUS overexpression. The LOTUS transgene was constructed by inserting HA-fused mouse *lotus* between the Igk chain secretion signal and the rabbit β-globin intron/polyA. The overexpression of the protein is driven by the synapsin-1 promoter (Fig. 1a). The purified plasmid was then microinjected into the pronuclei of fertilized oocytes derived from C57BL/6J mice. LOTUS overexpression was confirmed in heterozygous and homozygous LOTUS-TG mice (Fig. 1b,c). These mice were housed in a standard mouse facility and provided with autoclaved diet and water. Throughout the experimental procedures, all efforts were made to minimize the number of animals used and their suffering. The experimental procedures were approved by the institutional animal care and use ethical committee of Yokohama City University and were carried out in accordance with the approved guidelines. The *lotus/crtac1b* mutant was assessed on the C57BL/6J background.

#### Optic nerve crush model

Adult female C57BL/6 mice (20–25 g) were used to generate the optic nerve crush model. The mice were placed in a quiet and temperature- and humidity-controlled room (22 ± 3 °C and 60 ± 5%, respectively) in which a 12/12-hour light-dark cycle was maintained. All of the procedures here and throughout were reviewed by the Yale Institutional Animal Care and Use Committee (IACUC) and adhered to the procedures of the ARVO Statement for the Use of Animals in Ophthalmic and Vision Research.

### Antibodies

An affinity-purified polyclonal rabbit antibody against LOTUS (MBL, Nagoya, Japan) and an affinity-purified monoclonal mouse antibody against LOTUS (ITM, Matsumoto, Japan) were used for immunohistochemistry. The following antibodies were commercially obtained: affinity-purified polyclonal goat antibody against serotonin (#20079, 1:1000; Immunostar, Hudson, Wisconsin, USA); an affinity-purified polyclonal rabbit antibody against a synthetic peptide derived from residues 200 to the C-terminus of Rat GAP43, conjugated to KLH (#ab16053, Abcam, Milton, Cambridge, UK); an affinity-purified polyclonal rabbit antibody against a peptide mapping within an internal region of the influenza haemagglutinin (HA) protein (#sc-805, 1:500; Santa Cruz Biotechnology, Dallas, TX, USA); an affinity-purified monoclonal mouse antibody against native chick brain microtubules (#sc-32293, Santa Cruz Biotechnology, #); an affinity-purified polyclonal goat antibody against NgR1 (#AF1440, R&D, Minneapolis, MN, USA,); Alexa488-conjugated goat anti-rabbit IgG (#111-545-003, 1:500; Jackson ImmunoResearch, West Grove, PA, USA, West Grove, PA, USA); Alexa594-conjugated donkey anti-goat IgG (#705-585-147, 1:500; Jackson ImmunoResearch); horseradish peroxidase (HRP)-conjugated donkey anti-rabbit IgG (#NA934, 1:1000; GE Healthcare, Chicago, IL, USA); HRP-conjugated sheep anti-mouse IgG (#NA9310, 1:1000; GE Healthcare); and HRP-conjugated donkey anti-goat IgG (#705-035-147, 1:5000; Jackson ImmunoResearch).

### Reagents

Phosphate-buffered saline (137 mM NaCl, 2.68 mM KCl, 8.1 mM Na_2_VO_4_, 1.47 mM KH_2_PO_4_, pH 7.4; PBS, 0.3% Triton X-100; PBS-T), Tris-buffered saline (20 mM Tris-HCl (pH 7.6), 137 mM NaCl; TBS, 0.1% Tween-20; TBS-T), high-salt buffer (500 mM NaCl, 9.2 mM NaH_2_PO_4_, 12.5 mM Na_2_HPO_4_, 0.3% Triton X-100; HSB), and lysis buffer (20 mM Tris-HCl (pH 7.6), 150 mM NaCl, 1 mM EDTA-NaOH (pH 8.0), 1% Nonidet P-40, 1 mM Na_3_VO_4_, 0.05 mM *p*-amidinophenylsulfonyl fluoride, 0.1 U/mL aprotinin) were used.

### Animal Model of SCI

Adult female C57BL/6J mice (7–9 weeks old, weighing 18–25 g) were anaesthetized by the inhalation of isoflurane (Pfizer, New York, NY, USA). A laminectomy was performed between the 8^th^ and 9^th^ thoracic spinal vertebrae. The dura was cut with a micro-needle holder, and the dorsal over-hemitransection was then performed at the 8^th^ thoracic spinal ventral level to a depth of 1.0 mm. The 1.0-mm-deep lesion (dorsal over-hemitransection) was ensured by passing a 32-gauge needle through the lesion. The muscles and skin were then tightly sutured. After surgery, manual emptying of the bladder was performed every day to prevent bladder infections.

### Behavioural Tests

To determine functional recovery, we evaluated locomotion for 4 weeks using the BMS score, according to the original criteria^27^, and using footprints and the grid-walking test 4 weeks after SCI. The BMS open field test was scored 1, 3, 5, 7, 10, 14, 21, and 28 days after SCI to evaluate hind limb motor function, and locomotor activities were analysed using only mice showing scores of 1 at 1 day after SCI. Each mouse was observed for 1 min during each session. In the grid-walking test, the animals are required to cross a 30 × 30 cm horizontal wire grid with 1.0 × 1.0 cm square gaps. Grid-walking errors (foot slips below the grid) were counted from recorded videos. To examine the hind-limb stepping pattern during forward locomotion, the hind paws were coloured with ink to record footprints.

### Immunohistochemistry

After the last behavioural tests at 28 days after SCI, the mice were deeply anaesthetized with isoflurane (Pfizer) and sacrificed by transcardial perfusion with 25 ml of cold PBS, followed by 25 ml of 4% paraformaldehyde in cold PBS. After perfusion, a 10 mm-block of the spinal cord containing the injury site in the middle was carefully removed and post-fixed for several hours in the same fixative. Then, the sample blocks were successively transferred to 15% and 30% sucrose in PBS overnight. The spinal cords were embedded in Tissue-Tek OCT compound (Sakura Finetek, Tokyo, Japan) and kept frozen at −80 °C until use. Parasagittal sections of the spinal cord were cut at a thickness of 30 μm on a cryostat (#HM550-VPD, Thermo Scientific, Waltham, MA, USA) and mounted on glass slides (Matsunami, Osaka, Japan). To visualize 5-HT or HA immunostaining, the sections were washed three times with HSB or PBS-T and blocked with 5% normal horse serum in HSB or PBS-T for 30 min at room temperature (RT). Immunohistochemistry was then performed on the mounted sections with the indicated antibodies. The sections were incubated with anti-serotonin (Immunostar) or anti-HA (Santa Cruz Biotechnology) overnight at 4 °C, followed by Alexa594-labelled donkey anti-goat IgG (Jackson ImmunoResearch) or Alexa488-labelled goat anti-rabbit IgG (Jackson ImmunoResearch) antibodies for 1 hour at RT. To examine the morphological recovery of axonal projections, we evaluated the percentage of area over the 5-HT threshold and compared the rostral and caudal projections. The intensity of 5-HT immunostaining was analysed with MetaMorph software (Molecular Device Corp., Sunnyvale, CA, USA). To visualize myelin, a 5-mm block of spinal cord containing the injury site in the middle was carefully removed, and the blocks were frozen and processed as above. Parasagittal sections of the spinal cord were cut at 30-μm thickness on a cryostat (#HM550-VPD, Thermo Scientific). The sections were incubated with Luxol Fast Blue Stain Solution (Muto Pure Chemicals, Tokyo, Japan) overnight at 56 °C, cooled to RT, and then washed with 70% ethanol.

### Immunoblotting

To determine the change in LOTUS expression after SCI, the mice were anaesthetized by the inhalation of isoflurane (Pfizer) at 0, 1, 3, 5, 7, 10, 14, 21, and 28 days after SCI. Each sample lysate was prepared from a 3-mm block of the spinal cord containing the injury site in the middle. Proteins (20 μg per lane) were separated using sodium dodecyl sulfate polyacrylamide gel electrophoresis (8% gel) with a Laemmli buffer system. After electrophoretic transfer to polyvinylidene difluoride membranes (Millipore), non-specific binding was blocked with 5% skim milk in TBS-T for 1 h at RT, followed by incubation with an anti-LOTUS antibody (MBL, 1:2500) in 1% skim milk in TBS-T for 1 h at RT, followed by HRP-conjugated anti-rabbit IgG (GE Healthcare, 1:1000) for 1 h at RT. Enhanced Chemiluminescence Plus reagent (Merck Millipore, Darmstadt, Germany) was used to visualize immunostaining. For blot densitometry, the images of the protein bands were captured with the iQuant system, and band density was determined using an imager (ImageQuant TL, GE Healthcare).

### Intravitreal Injection, Optic Nerve Injury, and Anterograde Tracing

All of the experimental procedures were performed in compliance with animal protocols approved by the IACUC at Yale University School of Medicine (New Haven, CT). To express exogenous genes in retinal ganglion cells (RGCs), the mice were anaesthetized with ketamine and xylazine. One millilitre of adeno-associated virus-2 (AAV2-GFP or AAV2-HA-LOTUS) was injected intravitreally (titres at 1e^11^ TU/mL) in each eye. Care was taken during injections to avoid damage to the lens. Two weeks after viral injections, the optic nerves were exposed intraorbitally and crushed with jeweller’s forceps (#5–45, Fine Science Tools, Foster City, CA, USA) for 10 s approximately 1 mm behind the optic disc. Care was taken not to damage the tissue underneath the optic nerve during the crush. The completeness of each lesion was verified by anterograde tracing. Nineteen days after the crush, RGC axons were traced with 1 μL of Alexa555-labelled cholera toxin subunit-B (CTB) (2 μg/μL, Invitrogen, dissolved in sterile PBS) injected intravitreally in each eye. The animals were perfused 2 days after CTB injection.

### Histology, Imaging, and Quantification of the Optic Nerve Measurements

Optic nerves were dissected, straightened, and post-fixed in 4% paraformaldehyde overnight at 4 °C. Next, the nerves were optically cleared by incubating in a series of organic solvents: 50% tetrahydrofuran in distilled water (30 min), 80% tetrahydrofuran (30 min), 100% tetrahydrofuran (30 min, ×3), dichloromethane (20 min), and benzyl alcohol-benzyl benzoate (>15 min). Confocal stacks of each optic nerve were taken with a 20× objective on a Zeiss LSM 710 microscope with 4 μm optic sections. To quantify axon regeneration, vertical lines were drawn at fixed distances (200, 500, 1000, and 1500 µm) distal to the crush site, and the number of CTB-positive axons intersecting the vertical lines was counted on each section.

### Retrograde Labelling of Retinal Ganglion Cells

To identify RGCs, fluoro-gold (FG) was applied in the superior colliculi 7 days before the optic nerve crush as previously reported^30^ (n = 6). The mice were anaesthetized with 2% isoflurane in an oxygen–air mixture using a gas anaesthesia mask in a stereotaxic frame. An incision in the skin was made to expose the skull, and bregma was identified. Two holes (diameter = 1 mm) in the skull above the superior colliculus were drilled. The location of these holes was 2.9 mm behind bregma and 0.5 mm lateral the midline of each hemisphere. Cerebral tissue over the superior colliculus was aspirated gently. A thin layer of gelatine sponge pre-soaked with 6% of Fluro-Gold (Santa Cruz Biotechnology, Dallas, TX, USA) was filled into these drilled holes. The skin incision was sutured with 4-0 Vicryl. For FG-labelled RGCs counting, the flat-mounted whole retina was prepared and imaged as previously reported^31^. Briefly, the retina was divided into four equally sized quadrants. Four non-overlapping images were captured from the median line of each quadrant under a Zeiss LSM 710 confocal microscope. The total number of RGCs in 16 microscope fields per retina was counted by an automated procedure using the cell counting function of ImageJ (https://imagej.nih.gov; National Institutes of Health [NIH], Bethesda, MD, USA).

### Statistical Analysis

J-STAT software was used for statistical analysis. All of the data are expressed as the mean ± standard error (SE). The BMS score data were analysed with the Mann-Whitney U-test. The mean values of LOTUS and 5-HT protein levels, score data in the grid-walking test, and body weight were analysed with one-way ANOVA with the Tukey test. The expression levels of endogenous LOTUS, overexpressed HA-LOTUS and the beam walking time were analysed with Student’s t-test. Regenerating fibres in the optic nerve were analysed with two-way ANOVA with Fisher’s LSD post hoc test. Differences were considered significant at p < 0.05.

## Electronic supplementary material

Supplementary information
