# Supplementary material for: Regulation of axonal regeneration by the level of function of the endogenous Nogo receptor antagonist LOTUS
Source: Sci Rep. 2017 Sep 21;7:12119. doi: 10.1038/s41598-017-12449-6 (PMC5608707; doi:10.1038/s41598-017-12449-6)
Supplement: Supplementary file 1 — Supplementary information [file 41598_2017_12449_MOESM1_ESM.pdf]

Supplementary information

Title:

Regulation of axonal regeneration by the level of function of the endogenous Nogo receptor antagonist LOTUS

Authors:

Tomoko Hirokawa[1], Yixiao Zou[2], Yuji Kurihara[1], Zhaoxin Jiang[2], Yusuke Sakakibara[3], Hiromu Ito[3], Kengo Funakoshi[4], Nobutaka Kawahara[5,6], Yoshio Goshima[3,6], Stephen M. Strittmatter[2], and Kohtaro Takei[1,6, *]

1 Molecular Medical Bioscience Laboratory, Yokohama City University Graduate School of Medical Life Science, Yokohama 230-0045, Japan

2 Cellular Neuroscience, Neurodegeneration and Repair Program, Departments of Neurology and Neuroscience, Yale University School of Medicine, New Haven, CT 06536, USA

3 Department of Molecular Pharmacology and Neurobiology, Yokohama City University Graduate School of Medicine, Yokohama 246-0004, Japan

4 Department of Neuroanatomy, Yokohama City University Graduate School of Medicine, Yokohama 246-0004, Japan

5 Department of Neurosurgery, Yokohama City University Graduate School of Medicine, Yokohama 246-0004, Japan

6 Advanced Medical Research Center, Yokohama City University Graduate School of Medicine, Yokohama 236-0004, Japan

Corresponding author:

*Kohtaro Takei, Molecular Medical Bioscience Laboratory, Yokohama City University Graduate School of Medical Life Science, 1-7-29 Suehiro-cho, Tsurumi-ku, Yokohama

Supplementary Fig. S1

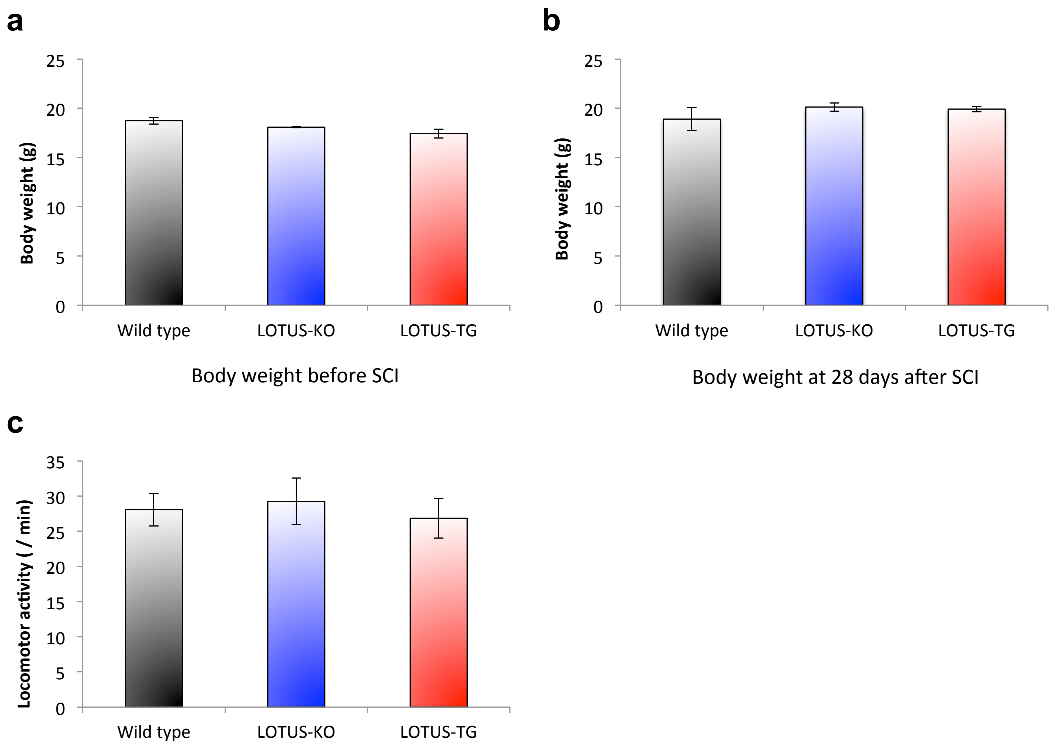

Supplementary Figure S1

Comparison of the body weight and open field locomotor activity in WT, LOTUS-KO, and LOTUS-TG mice. **a** and **b**, Body weight of WT, LOTUS-KO, and LOTUS-TG mice 0 (**a**) and 28 days (**b**) after SCI. No significant difference was observed with one-way ANOVA and the Tukey test (WT: n = 5; LOTUS-KO: n = 5; LOTUS-TG: n = 7). **c**, Open field activity was quantified by counting the number (per min) of line crossings divided into nine fractions in the field (35 × 25 cm). No significant difference was observed with one-way ANOVA and the Tukey test (WT: n = 13; LOTUS-KO: n = 5; LOTUS-TG: n = 16).

Supplementary Fig. S2

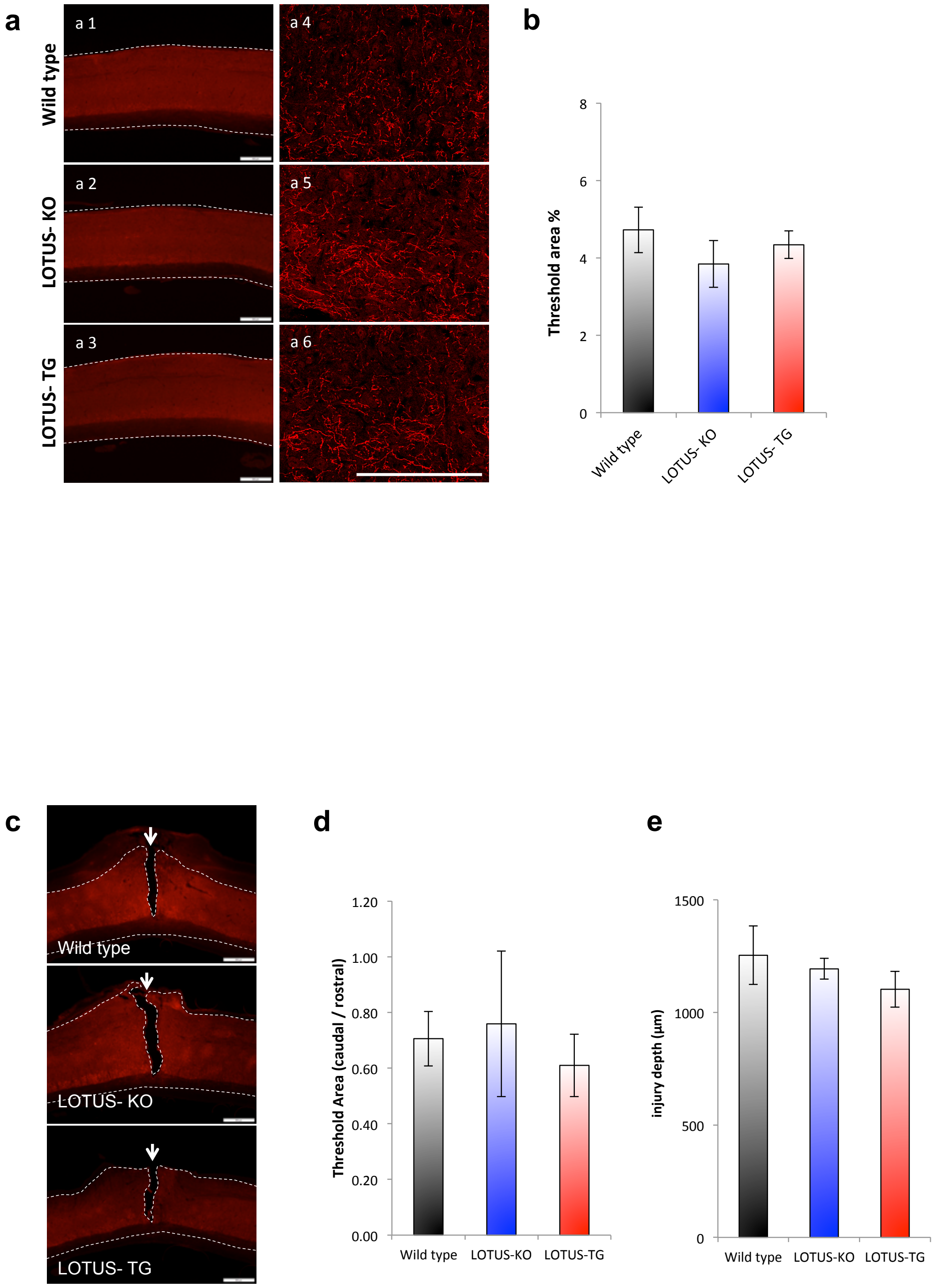

Supplementary Figure S2

Comparison of the 5-HT immunoreactivity and depth of injury site 1 day after SCI in WT, LOTUS-KO, and LOTUS-TG mice. (**a)** Images from sagittal sections of 5-HT immunohistochemical staining revealed 5-HT-positive raphespinal tract axons in each genotype of mice before SCI. Scale bar: 500 µm (a1- a3) and 200 µm (a4- a6). (**b)** Quantitative analysis of 5-HT immunoreactivity. No significant difference was observed using one-way ANOVA with the Tukey test (WT: n = 3; LOTUS-KO: n = 3; LOTUS-TG: n = 3). (**c)** Images from sagittal sections of 5-HT immunohistochemical staining revealed 5-HT-positive raphespinal tract axons caudal to the injury site in each genotype of mice 1 day after SCI. The arrows indicate the injury site. Scale bar: 500 µm. (**d)** Quantitative analysis of 5-HT immunoreactivity within 0.5 mm caudal to the injury site compared to that rostral to the injury site is shown as the relative ratio (caudal/rostral in each animal) of 5-HT immunoreactivity. No significant difference was observed using one-way ANOVA with the Tukey test (WT: n = 3; LOTUS-KO: n = 3; LOTUS-TG: n = 3). (**e)** The depth of injury site in each genotype of mice at 1 day after SCI. No significant difference was observed with one-way ANOVA and the Tukey test (WT: n = 3; LOTUS-KO: n = 2; LOTUS-TG: n = 3).

Supplementary Fig. S3

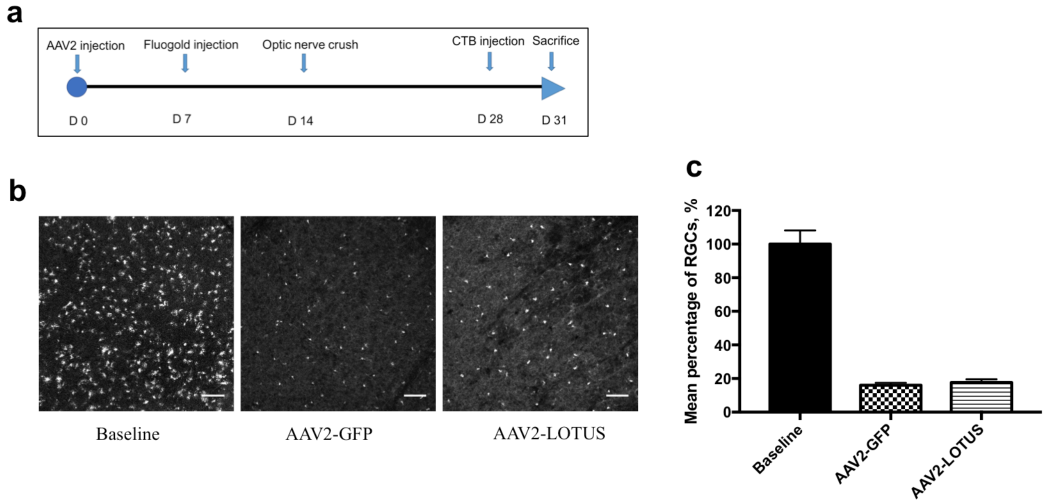

Supplementary Figure S3

LOTUS overexpression did not affect the survival of RGCs. (**a**) Fluoro-gold (FG) was applied in the superior colliculi 7 days before the optic nerve crush. (**b**) Representative images of FG-labelled RGCs in optic nerve-crushed eyes that received AAV-GFP or AAV-LOTUS treatment (bar = 50 μm). (**c**) The percentage of survival RGCs at 17 days post-optic nerve crush compared to baseline. An average of 17% of RGCs survived, and no significant difference was observed between the control and LOUTS overexpression groups ($P > 0.05$).

Supplementary Fig. S4

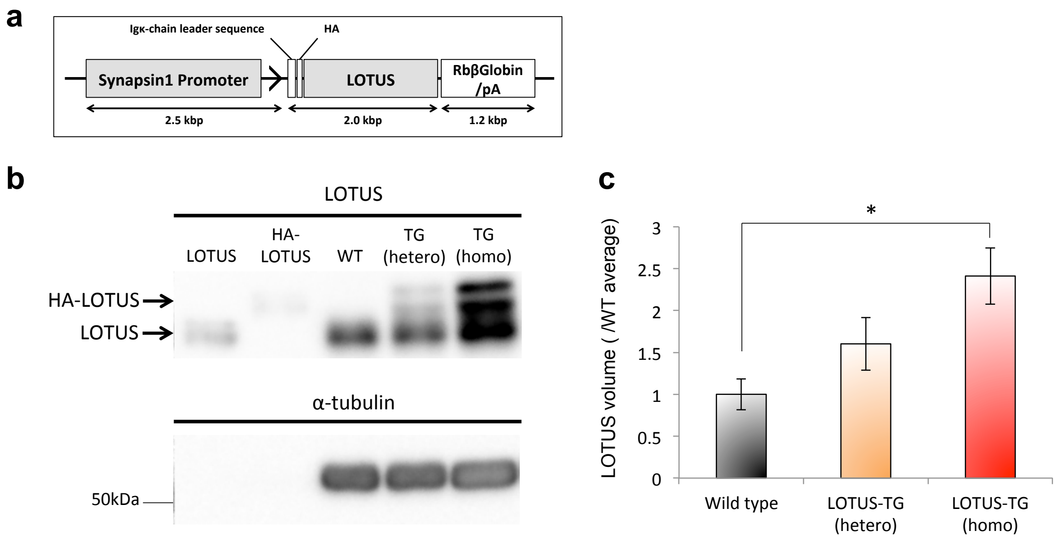

Supplementary Figure S4

(**a**) Schematic drawing of the construct used to generate transgenic mice overexpressing LOTUS. HA-tagged mouse LOTUS is composed of the mouse synapsin-1 promoter, the Igk-chain leader sequence, an HA tag, mouse *lotus* cDNA (except for the signal sequence), and the rabbit β-globin intron/polyA sequence. (**b**) Immunoblots showing LOTUS expression in the brains of WT, LOTUS-TG (heterozygous), and LOTUS-TG (homozygous) mice. (**c**) The expression level of overexpressed HA-LOTUS was quantified by the intensity of protein immunoblotting and normalized to the intensity of the WT band. Significance was obtained by performing one-way ANOVA with the Tukey test (n = 4 experiments). *$p < 0.05$ compared with the WT ratio.

Supplementary Fig. S5

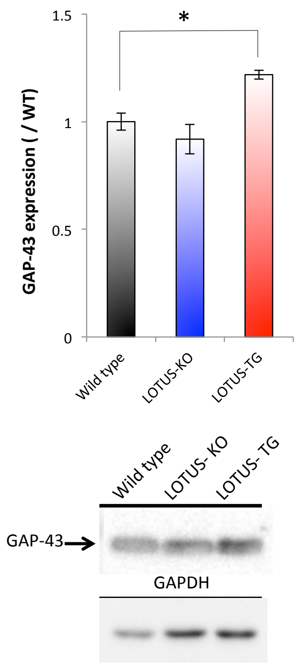

Supplementary Figure S5

Western blot analysis of GAP-43 expression in the injured region of spinal cord after SCI. Significance was analysed by performing one-way ANOVA with the Tukey test ($n = 3$ experiments). $^{*}p < 0.05$ compared with the WT.
